# Conditional quantum plasmonic sensing

**DOI:** 10.1515/nanoph-2022-0160

**Published:** 2022-06-15

**Authors:** Fatemeh Mostafavi, Zeinab Jafari, Michelle L. J. Lollie, Chenglong You, Israel De Leon, Omar S. Magaña-Loaiza

**Affiliations:** Quantum Photonics Laboratory, Department of Physics & Astronomy, Louisiana State University, Baton Rouge 70803, LA, USA; School of Engineering and Sciences, Tecnologico de Monterrey, Monterrey, Nuevo Leon 64849, Mexico

**Keywords:** particle subtraction, quantum measurement, quantum plasmonic sensing

## Abstract

The possibility of using weak optical signals to perform sensing of delicate samples constitutes one of the main goals of quantum photonic sensing. Furthermore, the nanoscale confinement of electromagnetic near fields in photonic platforms through surface plasmon polaritons has motivated the development of highly sensitive quantum plasmonic sensors. Despite the enormous potential of plasmonic platforms for sensing, this class of sensors is ultimately limited by the quantum statistical fluctuations of surface plasmons. Indeed, the fluctuations of the electromagnetic field severely limit the performance of quantum plasmonic sensing platforms in which delicate samples are characterized using weak near-field signals. Furthermore, the inherent losses associated with plasmonic fields levy additional constraints that challenge the realization of sensitivities beyond the shot-noise limit. Here, we introduce a protocol for quantum plasmonic sensing based on the conditional detection of plasmons. We demonstrate that the conditional detection of plasmonic fields, via plasmon subtraction, provides a new degree of freedom to control quantum fluctuations of plasmonic fields. This mechanism enables improvement of the signal-to-noise ratio of photonic sensors relying on plasmonic signals that are comparable to their associated field fluctuations. Consequently, the possibility of using weak plasmonic signals to sense delicate samples, while preserving the sample properties, has important implications for molecule sensing, and chemical detection.

The possibility of controlling the confinement of plasmonic near-fields at the subwavelength scale has motivated the development of a variety of extremely sensitive nanosensors [1–4]. Remarkably, this class of sensors offers unique resolution and sensitivity properties that cannot be achieved through conventional photonic platforms in free space [4–7]. In recent decades, the fabrication of metallic nanostructures has enabled the engineering of surface plasmon resonances to implement ultrasensitive optical transducers for detection of various substances ranging from gases to biochemical species [1, 2, 4]. Additionally, the identification of the quantum mechanical properties of plasmonic near-fields has prompted research devoted to exploring mechanisms that boost the sensitivity of plasmonic sensors [8–12].

The scattering paths provided by plasmonic near-fields have enabled robust control of quantum dynamics [12–15]. Indeed, the additional degree of freedom provided by plasmonic fields has been used to harness the quantum correlations and quantum coherence of photonic systems [12, 14, 16, 17]. Similarly, this exquisite degree of control made possible the preparation of plasmonic systems in entangled and squeezed states [18–21]. Among the large variety of quantum states that can be engineered in plasmonic platforms [10, 11], entangled systems in the form of N00N states or in diverse forms of squeezed states have been used to develop quantum sensors [4, 22], [23], [24], [25]. In principle, the sensitivity of these sensors is not constrained by the quantum fluctuations of the electromagnetic field that establish the shot-noise limit [7, 26]. However, due to inherent losses of plasmonic platforms, it is challenging to achieve sensitivities beyond the shot-noise limit under realistic conditions [5]. Despite existing obstacles, recent work demonstrates the potential of exploiting nonclassical properties of plasmons to develop quantum plasmonic sensors for detection of antibody complexes, single molecules, and to perform spectroscopy of biochemical substances [27–30].

Here, we explore a new scheme for quantum sensing based on plasmon-subtracted thermal states [31–33]. Our work offers an alternative to quantum sensing protocols relying on entangled or squeezed plasmonic systems [4, 18], [19], [20], [21], [22], [23], [24], [25]. We use a sensing architecture based on a nanoslit plasmonic interferometer [34]. It provides a direct relationship between the light exiting the interferometer and the phase shift induced in one of its arms by the substance to be sensed (analyte). We introduce a conditional quantum measurement on the interfering plasmonic fields via the subtraction of plasmons. We show that this process enables the reduction of quantum fluctuations of the sensing field and increases the mean occupation number of the plasmonic sensing platform [32, 33]. Furthermore, plasmon subtraction provides a method for manipulating the signal-to-noise ratio (SNR) associated with the measurement of phase shifts. We demonstrate that the reduced fluctuations of plasmonic fields lead to an enhancement in the estimation of a phase shift. The performance of our protocol is quantified through the uncertainty associated to phase measurements. We point out that the reduced uncertainties in the measurement of phases leads to better sensitivities of our sensing protocol. This study is conducted through a quantum mechanical model that considers the realistic losses that characterize a plasmonic nanoslit sensor. We report the probabilities of successfully implementing our protocol given the occupation number of the plasmonic sensing fields and the losses of the nanostructure. Our analysis suggests that our protocol offers practical benefits for lossy plasmonic sensors relying on weak near-field signals [35]. Consequently, our platform can have important implications for plasmonic sensing of delicate samples such as molecules, chemical substances or, in general, photosensitve materials [27–30].

We first discuss the theoretical model that we use to describe conditional quantum measurements applied to a thermal plasmonic system. Figure 1a describes the interactions supported by the plasmonic nanoslit under consideration [36]. This nanostructure acts as a plasmonic tritter by coupling the photonic mode $\hat{b}$ and the two plasmonic modes, described by the operators $\hat{a}$ and $\hat{c}$ , to three output modes [36]. The photonic mode at the output of the nanoslit is described by $\hat{e}$ , whereas the two plasmonic output modes are represented by the operators $\hat{f}$ and $\hat{d}$ . As indicated in Figure 1b, and throughout this paper, we study the conditional detection of the output modes $\hat{d}$ and $\hat{e}$ for a situation in which only the input plasmonic modes of $\hat{a}$ and $\hat{c}$ are excited in the nanostructure. Thus, the photonic mode $\hat{b}$ is assumed to be in a vacuum state. In this case, the plasmonic tritter can be simplified to a two-port device described by the following 2 × 2 matrix.

(1) $$\left(\begin{array}{c} \hat{d}\\ \hat{e} \end{array}\right)=\left(\begin{array}{cc} \kappa & r\\ \tau & \tau \end{array}\right)\left(\begin{array}{c} \hat{a}\\ \hat{c} \end{array}\right).$$

**Figure 1:**
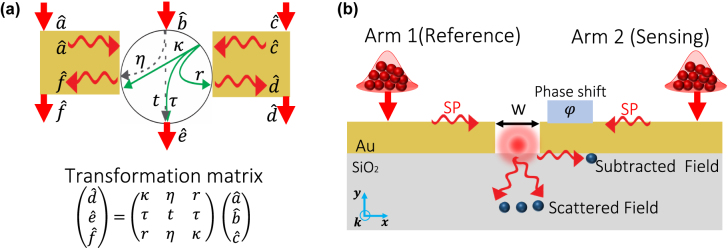
Optical interactions in the plasmonic nanoslit and its use for conditional quantum sensing. (a) Schematic diagram of the interactions in a plasmonic nanoslit. The plasmonic nanostructure has three input and three output ports. The photonic mode at the input is described by the operator $\hat{b}$ , whereas the two plasmonic modes are represented by $\hat{a}$ and $\hat{c}$ . These modes are coupled to the plasmonic modes $\hat{d}$ and $\hat{f}$ , and to the photonic mode $\hat{e}$ at the output of the nanostructure. As described by the transformation matrix, the parameters *κ*, *τ*, *r*, *η*, and *t* represent the coupling coefficients among the ports of the nanostructure. For sake of clarity, the diagram only illustrates the coupling paths for the input modes $\hat{b}$ and $\hat{c}$ . The diagram in (b) shows the design of our simulated plasmonic sensor, comprising a slit of width w in a 200 nm gold thin film. Here, the plasmonic structure is illuminated by two thermal multiphoton sources that excite two plasmonic fields with super-Poissonian statistics (the input grating couplers are not shown in the figure). The two counter-propagating surface plasmon (SP) modes interfere at the interface between the gold layer and the SiO_2_ substrate. The interference conditions are defined by the phase shift *φ* induced in one of the plasmonic modes by the substance that we aim to sense.

The photonic mode $\hat{e}$ is transmitted through the slit and its transmission probability is described by 2|*τ*|^2^ = *T*_ph_. Here, *T*_ph_ represents the normalized intensity of the transmitted photons. Moreover, the plasmon-to-plasmon coupling at the output of the nanostructure is given by |*κ*|^2^ + |*r*|^2^ = *T*_pl_. Here, the renormalized transmission (after intereference and considering loss) for the plasmonic fields is described by *T*_pl_. From Figure 1b, we note that the interference supported by the plasmonic nanoslit shares similarities with those induced by a conventional Mach–Zehnder interferometer (MZI). More specifically, the two plasmonic modes, $\hat{a}$ and $\hat{c}$ , interfere at the location of the nanoslit, which in turn scatters the field to generate the output [34]. The interference conditions are defined by the phase shift induced by the analyte. Plasmonic sensors with nanoslits have been extensively investigated in the classical domain, showing the possibility of ultrasensitive detection using minute amounts of analyte [1], [2], [3], [4, 23, 34].

We now consider a situation in which a single-mode thermal light source is coupled to the nanostructure in Figure 1b exciting two counter-propagating surface plasmon modes. This can be achieved by using a pair of grating couplers (not shown in the figure) [34]. The statistical properties of this thermal field can be described by the Bose–Einstein statistics as $\rho_{\text{th}}=\sum_{n=0}^{\infty }p_{\text{pl}}\left(n\right)|n〉〈n|$ , where $p_{\text{pl}}\left(n\right)={\bar{n}}^{n}/{\left(1+\bar{n}\right)}^{1+n}$ , and $\bar{n}$ represents the mean occupation number of the field. Interestingly, the super-Poissonian statistics of thermal light can be modified through conditional measurements [31–33]. As discussed below, it is also possible to modify the quantum statistics of plasmonic fields. The control of plasmonic statistics can be implemented by subtracting/adding bosons from/to thermal plasmonic systems [37, 38]. In this work, we subtract plasmons from the transmitted field formed by the superposition of the surface plasmon modes propagating through the reference and sensing arms of the interferometer. This transmitted mode $\hat{e}$ is then conditioned to the output of the field $\hat{d}$ . As such, the number of subtracted particles *L* is experimentally controlled by the strength of the plasmonic near fields surrounding our sensor, and the conditional counting of particles in mode $\hat{d}$ . The strength of the near field is defined by the design of the plasmonic nanostructure, whereas the conditional counting is experimentally implemented by measuring simultaneous detection events between modes $\hat{d}$ and $\hat{e}$ . It is worth mentioning that conditional measurements in photonic systems have been experimentally demonstrated in Refs. [32, 33]. The successful subtraction of plasmons boosts the signal of the sensing platforms. This feature is particularly important for sensing schemes relying on dissipative plasmonic platforms.

The conditional subtraction of *L* plasmons from the mode $\hat{d}$ leads to the modification of the quantum statistics of the plasmonic system, this can be described by

(2) $$p_{\text{pl}}\left(n\right)=\frac{\left(n+L\right)!{\bar{n}}_{\text{pl}}^{n}}{n!L!{\left(1+{\bar{n}}_{\text{pl}}\right)}^{L+1+n}},$$

where ${\bar{n}}_{\text{pl}}$ represents the mean occupation number of the quasi-particles that constitute the scattered field in mode $\hat{e}$ (see Supplementary Material). We quantify the modification of the quantum statistics through the degree of second-order correlation function *g* ^(2)^(0) for the mode $\hat{e}$ as (see also Supplementary Material)

(3) $$g_{L}^{\left(2\right)}\left(0\right)=\frac{L+2}{L+1}.$$

We note that the conditional subtraction of plasmons induces anti-thermalization effects that attenuate the fluctuations of the plasmonic thermal system used for sensing. Indeed, the $g_{L}^{\left(2\right)}\left(0\right)$ approaches one with the increased number of subtracted plasmons, namely large values of *L*. This effect produces bosonic distributions resembling those of coherent states [32]. Recently, similar anti-thermalization effects have been explored in photonic lattices [39].

The aforementioned plasmon subtraction can be implemented in the plasmonic nanoslit interferometer shown in Figure 1b. It consists of a 200 nm thick gold film deposited on a glass substrate [34]. This thickness is large enough to enable decoupled plasmonic modes on the top and bottom surfaces of the film, as required. The gold film features a 320 nm slit, defining the reference arm of the interferometer to its left and the sensing arm (holding the analyte) to its right. The analyte then induces a phase difference *φ* relative to the reference arm, thereby creating the output ( $\hat{d},\hat{e}$ and $\hat{f}$ ) that depends on this parameter.

We note that we aim to detect two plasmonic modes $\hat{d}$ and $\hat{e}$ in the far-field plane of the sample. This detection can be achieved using similar setups to those reported in [13, 40, 41]. Consequently, the experimental realization of this protocol includes two output ports in the form of grating couplers placed on the bottom surface of the gold film. These gratings are used to out-couple the $\hat{d}$ and $\hat{e}$ plasmonic modes into photons propagating in the substrate in the negative *y*-direction. Then, an optical system is used to collect the out-coupled photons from each port and direct them into two single-photon detectors. The photon-number-resolving detection of the scattered field can be performed through transition-edge sensors [42] or through the use of surjective photon counting techniques [33, 43]. This scheme has been extensively used in previous quantum plasmonic experiments such as those listed in Refs. [13, 40, 41]. To verify the feasibility of our conditional measurement approach, we perform a finite-difference time-domain (FDTD) simulation of the plasmonic nanoslit using a wavelength of *λ* = 810 nm for the two counter-propagating surface plasmon modes ( $\hat{a}$ and $\hat{c}$ ). The nanoslit is designed to support two localized surface plasmon (LSP) modes, one with dipolar symmetry and other with quadrupolar symmetry. Depending on the phase difference *φ*, these two LSP modes can be excited with different strengths by the fields interfering at the nanoslit being the dipolar (quadrupolar) mode optimally excited with *φ* = 0 (*φ* = *π*). This is due to the fact that the near-field symmetries of the interfering field are well-matched to the dipolar and quadrupolar fields for those values of *φ* [34]. Figure 2, panels (a) to (c), represent the near-field intensity distribution *I* of our designed nanoslit in the *x*–*y* plane. Panels (d) to (f) show the normalized near-field interference pattern produced by the field transmitted by a 320-nm-wide slit. These near-field interference patterns are associated with the excited LSP mode corresponding to *φ* = 0, *φ* = *π*/2, and *φ* = *π*. In this case, (d) and (f) illustrate the dipolar and quadrupolar modes of the nanostructure. The dashed lines in Figure 3 indicate the far-field angular distributions of the transmitted intensity associated with the dipolar LSP mode (panels a to d) and the quadrupolar LSP mode (panels i to l). Only a small angular range of the far-field distribution (range within vertical lines in Figure 3) is used as the sensing signal. Thus, the sensing signal varies monotonically from a maximum value at *φ* = 0 to a minimum value at *φ* = *π* [34]. The transmission parameters of our sensor are estimated from FDTD simulations. Specifically, the transmission values for the photonic and plasmonic modes are *T*_ph_ ≈ 0.076 and *T*_pl_ ≈ 0.0176 for *φ* = *π*. However, our subtraction scheme is general and valid for any phase angle *φ* in the range of 0 ≤ *φ* ≤ 2*π*. Moreover, the total amount of power coupled to modes $\hat{e}$ and $\hat{d}$ normalized to the input power of the plasmonic structure is defined as *γ* = *T*_ph_ + *T*_pl_ ≈ 0.0941. For the results shown in Figure 3, we assume a mean occupation number of $\bar{n}=3.75$ for the input beam. As shown in panels (a), (e) and (i) of Figure 3, the output signals, calculated from Eq. (2) and represented by the red shaded region across all panels, exhibit strong quantum fluctuations. Surprisingly, after performing plasmon subtraction, the quantum fluctuations decrease, as indicated in the panels (b)–(d), (f)–(h) and (j)–(l) of Figure 3. Evidently, this confirms that our conditional measurement protocol can indeed boost the output signal and consequently improve the sensing performance of a plasmonic device. However, due to the probabilistic nature of our protocol and the presence of losses, it is important to estimate the probability rates of successfully performing plasmon subtraction. Furthermore, we note that the structural parameters of the nanostructure will modify the scattering conditions among photons and plasmons. Consequently, the change of the structural parameters of our plasmonic nanoslit will also modify the probability of plasmon subtraction. In Table 1, we list the degree of second-order correlation $g_{L}^{\left(2\right)}\left(0\right)$ , and the probability of successfully subtracting one, two, and three plasmons for different occupation numbers of the plasmonic fields used for sensing.

**Figure 2:**
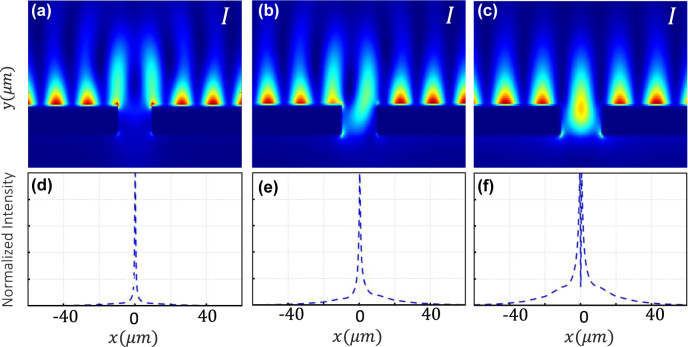
Panels (a–c) illustrate the intensity *I* of the near-field distribution of our designed nanoslit in the *x*–*y* plane. The blue dashed line in panels (d–f) show the normalized near-field interference pattern produced by the field transmitted by a 320-nm-wide slit. This corresponds to the scattered field described by mode $\hat{e}$ . The plots are obtained for *φ* = 0, *φ* = *π*/2, and *φ* = *π*, respectively.

**Figure 3:**
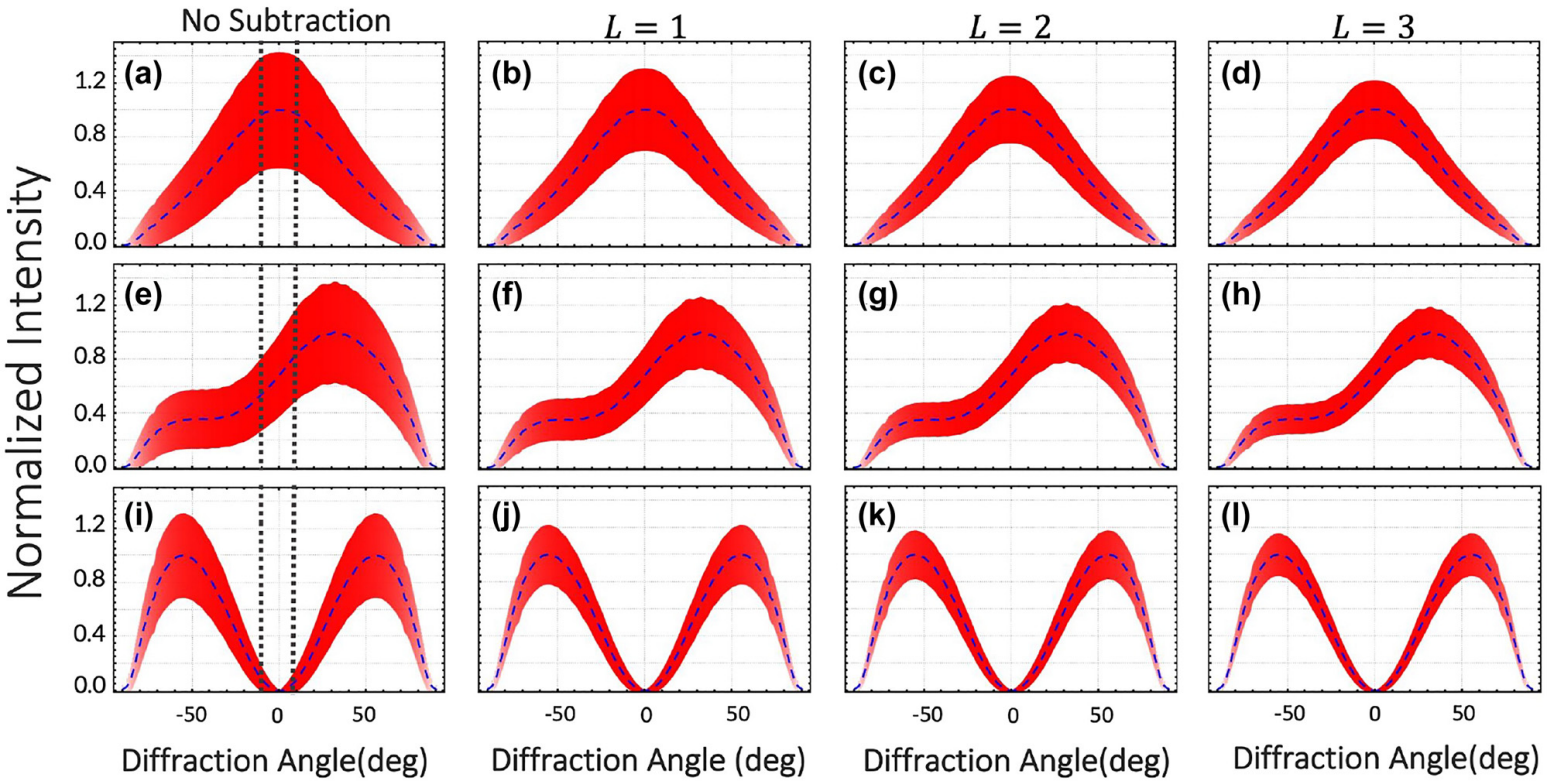
Normalized far-field intensity distribution scattered by the plasmonic nanoslit. The blue dashed line indicates the interference pattern produced by the field transmitted through the 320-nm-wide slit, this corresponds to mode $\hat{e}$ . The panels from (a) to (d) are obtained for *φ* = 0, whereas those from (e) to (h) and (i) to (l) are calculated for *φ* = *π*/2 and *φ* = *π*, respectively. The dashed line in all plots represents the intensity distribution of the fields transmitted through the slit indicative of dipolar and quadrupolar near-field symmetry for *φ* = 0 and *φ* = *π*. The red shaded regions correspond to the standard deviation for $\bar{n}=3.75$ . Panels (a), (e) and (i) depict the unconditional detection of the signal with its associated noise. As displayed in panels (b) to (d), (f) to (h) and (i) to (l), the signal-to-noise ratio of the plasmonic sensor improves as the fluctuations of the field are reduced through the conditional detection of plasmons. The vertical lines on panels (a), (e) and (i) represent the angular range used for the calculation of the intensity variation with phase (i.e. sensitivity depicted in Figure 4b).

**Table 1:** The estimated probability of plasmon subtraction and the corresponding degree of second-order coherence $g_{L}^{\left(2\right)}\left(0\right)$ .

$\bar{n}$	*L* = 1	*L* = 2	*L* = 3
2	1.0 × 10^−2^	1.0 × 10^−4^	1.1 × 10^−6^
1	5.2 × 10^−3^	2.7 × 10^−5^	1.4 × 10^−7^
0.5	2.6 × 10^−3^	7.0 × 10^−6^	1.8 × 10^−8^
0.3	1.5 × 10^−3^	2.5 × 10^−6^	4.0 × 10^−9^
$g_{L}^{\left(2\right)}\left(0\right)$	1.5	1.33	1.25

The losses of the plasmonic nanostructure reduce the probability of subtracting multiple plasmons *L* from the scattered field with an occupation number of $\bar{n}$ . In this case, we assume *φ* = *π*.

The quantities reported in Table 1 were estimated for a phase shift given by *φ* = *π*. This table considers realistic parameters for the losses associated to the propagation of the plasmonic sensing field, and the limited efficiency *η*_ph_ and *η*_pl_ of the single-photon detectors used to collect photonic and plasmonic mode, respectively. In this case, we assume *η*_ph_ = 0.3 and *η*_pl_ = 0.3, which corresponds to the efficiency of commercial single-photon detectors [33, 44]. In general, the value for *φ* determines how strongly the dipolar and quadrupolar LSP modes are excited, and consequently their far-field angular distributions. However, the process is applicable for other phases *φ*. Our predictions suggest that plasmonic subtraction can be achieved at reasonable rates using a properly designed nanostructure.

We now quantify the performance of our conditional scheme for plasmonic sensing through the SNR associated to the estimation of a phase shift. The SNR is estimated as the ratio of the mean occupation number to its standard deviation. This is defined as (see Supplementary Material)

(4) $$\text{SNR}=\sqrt{\frac{\left(1+L\right)\bar{n}\gamma \eta_{\text{ph}}\xi \;\cos^{2}\frac{\varphi }{2}}{1+\bar{n}\gamma \left(\xi \eta_{\text{ph}}+\left(1-\xi \right)\eta_{\text{pl}}\right)\cos^{2}\frac{\varphi }{2}}}.$$

Here, the parameter *ξ* = *T*_ph_/(*T*_ph_ + *T*_pl_) = 0.80 represents the normalized transmission of the photonic mode. In Figure 4a, we report the increasing SNR of our plasmonic sensor through the process of plasmon subtraction by plotting the SNR for the subtraction of one, two, and three plasmons for different phase shifts *φ*. In addition, for sake of completeness, we evaluate the improvement in sensitivity using error propagation [45]. More specifically, we calculate the uncertainty of a phase measurement Δ*φ*. This parameter is estimated as

(5) $$\Delta \varphi =\sqrt{\left\langle {\hat{n}}^{2}\right\rangle -{\left\langle \hat{n}\right\rangle }^{2}}/\left|\frac{d\left\langle \hat{n}\right\rangle }{d\varphi }\right|$$

**Figure 4:**
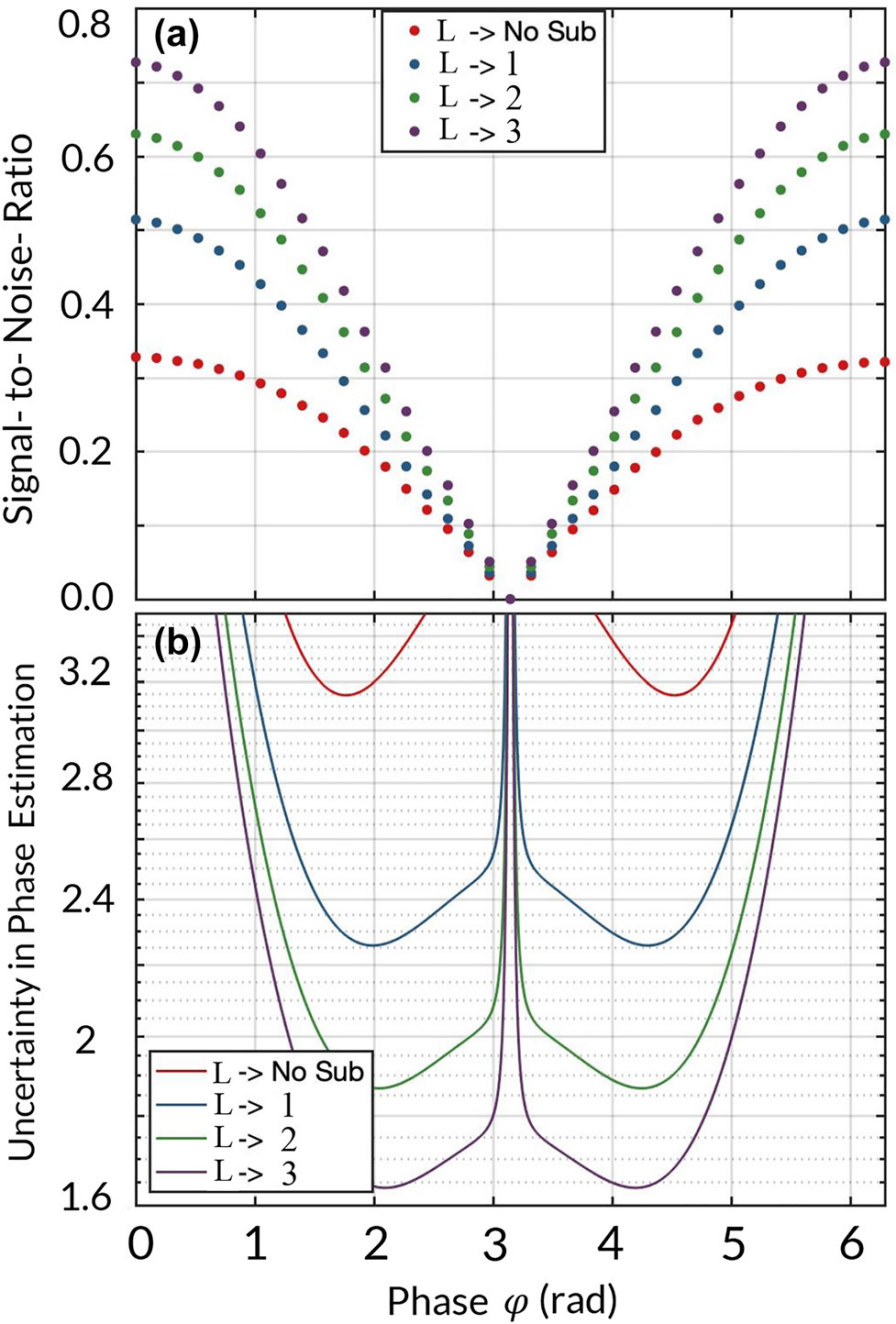
The panel in (a) reports the signal-to-noise ratio (SNR) as a function of *φ* for the conditional detection of the plasmonic modes transmitted by a 320-nm nanoslit. The red dots represent the unconditional SNR. Furthermore, the blue, green, and purple dots indicate the SNR for the subtraction of one, two, and three plasmons, respectively. This plot shows the possibility of improving the SNR of our plasmonic sensor through the subtraction of plasmons. The panel in (b) indicates that an increasing SNR leads to lower uncertainties in the estimation of phase shifts induced by analytes. The lower uncertainties described by Δ*φ* imply higher sensitivities of our plasmonic sensor.

The observable $\hat{n}={\hat{e}}^{†}\hat{e}$ , corresponds to the conditional intensity measurement within an angular range of the far-field distribution (specified in Figure 3 with the vertical lines). In the field of quantum metrology, the reduced uncertainty of a phase measurement Δ*φ* is associated to an improvement in the sensitivity of a quantum sensor [46, 47]. In this regard, the conditional detection of plasmons increases the sensitivity of our plasmonic sensor. This enhancement is reported in Figure 4b. Here, we demonstrate that the attenuation of the fluctuations of a weak plasmonic field, through the subtraction of up to three plasmons, leads to lower uncertainties in the sensing of photosensitive analytes.

We point out that our conditional measurement scheme is general and can be applied to any plasmonic sensing platform [48]. As demonstrated through Figure 4 and Eq. (4), the subtraction of plasmons boosts the signal-to-noise ratio of any electromagnetic field used for classical sensing. Thus, if a small physical parameter can be sensed by a classical scheme for plasmonic sensing, our technique will increase its signal-to-noise ratio. However, if the sample of interest acting as a phase shifter induces a change in the particle number that is smaller or equal than the statistical fluctuations (Δ*n*) of the sensing field, our technique cannot provide any advantage. In this situation, the signal cannot be discriminated from the inherent noise of the plasmonic near fields. As such, our scheme is capable of improving current capabilities for single-molecule detection if the signal induced by the single-molecule is stronger than the fluctuations of the sensing field [48].

In conclusion, we have investigated a new method for quantum plasmonic sensing based on the conditional subtraction of plasmons. We have quantified the performance of this scheme, under realistic conditions of loss, by considering the design of a real plasmonic nanoslit sensor. We showed that conditional measurements offer an important path for controlling the statistical fluctuations of plasmonic fields for sensing. In our work, we considered the case for which the sensing field contains a mean plasmonic number lower than four. In this regime, we showed that the attenuation of the quantum fluctuations of plasmonic fields increases the mean occupation number of the sensing field. Interestingly, this effect leads to larger signal-to-noise ratios of our sensing protocol. Furthermore, this feature of our technique enables performing sensitive plasmonic sensing with weak signals [1–4]. We believe that our work offers an alternative approach to boost signals in quantum plasmonic platforms operating in the presence of loss at the few particle regime [10, 11].

## Supplementary Material

Supplementary Material Details
