# Supplementary material for: Conditional quantum plasmonic sensing
Source: Nanophotonics. 2022 Jun 15;11(14):3299–306. doi: 10.1515/nanoph-2022-0160 (PMC11501117; doi:10.1515/nanoph-2022-0160)
Supplement: Supplementary file 1 — Supplementary Material Details [file j_nanoph-2022-0160_suppl.pdf]

Supplementary Material: Conditional Quantum Plasmonic Sensing

Fatemeh Mostafavi,[1] Zeinab Jafari,[2] Michelle L. J. Lollie,[1]
Chenglong You,[1] Israel De Leon,[2] and Omar S. Magaña-Loaiza[1]

[1] *Quantum Photonics Laboratory, Department of Physics & Astronomy, Louisiana State University, Baton Rouge, LA 70803, USA*
[2] *School of Engineering and Sciences, Tecnologico de Monterrey, Monterrey, Nuevo Leon 64849, Mexico*

In this supplementary material we present: (i) the details of FDTD simulation; (ii) the derivation of the degree of second-order coherence, particle statistics, and conditioned signal-to-noise ratio.

FDTD Simulation

The design of the plasmonic structure given in Fig. 1b is simulated with a 2-D FDTD simulations by a 130 μm domain in x direction and 8 μm along the y direction. The boundary condition is satisfied via the perfect matching layers to efficiently absorb the light scattered by the structure. Besides, the simulations time was long enough so that all energy in the simulation domain was completely decayed. The upper clad is made of CYTOP, a polymer with refractive index that closely matches the refractive index of 1.33. The mesh size was as small as 0.03 nm along x direction and where we have highly confined field propagation. To create the propagating plasmonic modes, we use a pair of mode sources in both sides of the central slit. The generated SP modes propagate toward the central slit where they interfere. The near-fields along a linear line underneath the nanostructure were extracted and used for the far-field analysis. The coupled light to the mode $\hat{e}$, i.e. T_{ph}, was calculated by the power flow through to the same linear line beneath the slit normalized to the input power. To have a realistic estimation of the subtracted light, the mode $\hat{d}$ was first propagated for a distance of 10λ (8.1 μm) along the gold-glass interface and then a grating coupling efficiency of 36% was considered to out couple the plasmonic mode to the free space [1]. The out-coupling was done far from the slit to avoid interactions of slit near-fields with fields of the assumed grating.

Derivation of the degree of second-order coherence, particle statistics, and conditioned signal-to-noise ratio

First, we calculate the second-order correlation function $g_L^{(2)}(0)$ associated to the L-plasmon-subtracted light field. We assume a thermal light field with Bose-Einstein statistics described by $\rho_{\text{th}} = \sum_{n=0}^{\infty} \text{p}_{\text{pl}}(n)|n\rangle\langle n|$, where $\text{p}_{\text{pl}}(n) = \bar{n}^n/(1+\bar{n})^{1+n}$. The subtraction of L-plasmon(s) from a single-mode thermal field gives

$$\rho_L = \frac{(\hat{a})^L \rho\, (\hat{a}^+)^L}{\text{Tr}\left((\hat{a})^L \rho\, (\hat{a}^+)^L\right)} = \sum_{n=0}^{\infty} \frac{(n+L)!}{n!L!} \frac{\bar{n}^n}{(1+\bar{n})^{L+n+1}} |n\rangle\langle n| = \text{p}_{\text{pl}}(n)|n\rangle\langle n|. \tag{S.1}$$

The second-order correlation function of a single-mode field is given by

$$g^{(2)}(0) = \frac{\langle \hat{a}^\dagger \hat{a}^\dagger \hat{a}\hat{a}\rangle}{\langle \hat{a}^\dagger \hat{a}\rangle^2} = \frac{\langle \hat{n}(\hat{n}-1)\rangle}{\langle \hat{n}\rangle^2} = \frac{\langle \hat{n}^2\rangle - \langle \hat{n}\rangle}{\langle \hat{n}\rangle^2}. \tag{S.2}$$

We can now calculate each element in Eq. (S.2). We have

$$\langle \hat{n}^2\rangle = \sum_{n=0}^{\infty} n^2 \text{p}_{\text{pl}}(n) = (L+1)\bar{n}[(L+2)\bar{n}+1]. \tag{S.3}$$

Similarly,

$$\langle \hat{n}\rangle = \sum_{n=0}^{\infty} n \text{p}_{\text{pl}}(n) = (L+1)\bar{n}. \tag{S.4}$$

Combining Eq. (S.2), Eq. (S.3) and Eq. (S.4), we obtain

$$g_L^{(2)}(0) = \frac{L+2}{L+1}, \tag{S.5}$$

which is independent of the mean occupation number $\bar{n}$ of the input thermal field.

Now we derive Eq. (5) in the main paper. First, we note that in our calculation, we assume that mode $\hat{a}$ and mode $\hat{c}$ come from the same input source. Following similar approaches to those presented in [2], for the lossless case, the mean occupation number of mode $\hat{e}$ is given by $\bar{n}_e = \bar{n}\xi\cos^2\left(\frac{\varphi}{2}\right)$. Here, $\bar{n}$ is the mean occupation number in the input modes $\hat{a}$ and $\hat{c}$, and ξ represents the normalized transmission of the plasmonic tritter [3]. However, we need to consider that the plasmonic structure induces loss, and we have non-unity detection efficiency. As discussed in the main body of the paper, conditional measurements will change the mean occupation number of the mode $\hat{e}$. We first consider the situation in which no plasmons are subtracted (no conditional measurement is implemented). In this case, the average occupation number of mode $\hat{e}$ is simply modulated by the loss γ of the plasmonic tritter, and the quantum efficiency η_{ph} of the detector,

$$\bar{n}_e = \bar{n}\gamma\xi\eta_{\mathrm{ph}}\cos^2\left(\frac{\varphi}{2}\right). \tag{S.6}$$

In this case, since no conditional measurement is made, the particle statistics are preserved. Therefore, the standard deviation is the same to that of a thermal field,

$$\Delta n_e = \sqrt{\bar{n}_e + \bar{n}_e^2}. \tag{S.7}$$

Therefore, the signal-to-noise ratio (SNR) is given by

$$\mathrm{SNR} = \frac{\bar{n}_e}{\Delta n_e} = \frac{\bar{n}_e}{\sqrt{\bar{n}_e + \bar{n}_e^2}} = \frac{\sqrt{\bar{n}\gamma\xi\eta_{\mathrm{ph}}\cos^2\left(\frac{\varphi}{2}\right)}}{\sqrt{\left(1 + \bar{n}\gamma\xi\eta_{\mathrm{ph}}\cos^2\left(\frac{\varphi}{2}\right)\right)}}. \tag{S.8}$$

Now we consider the conditional subtraction of plasmons. The L-plasmon subtracted state $\rho_e(L)$ of mode $\hat{e}$ is conditioned on detection of L plasmon(s) in mode $\hat{d}$ [4],

$$\rho_e(L) = \frac{1}{\mathrm{p}_d(L)}\mathrm{Tr}_{\mathrm{d}}\left[\rho\mathbb{I}\otimes\Pi_L\left(\eta_{\mathrm{pl}}\right)\right]. \tag{S.9}$$

Specifically, $\mathrm{p}_d(L)$ is the probability of measuring L plasmon(s) in mode $\hat{d}$. Since the transformation of the plasmonic tritter preserves the particle statistics, mode $\hat{d}$ still possess thermal statistics,

$$\mathrm{p}_d(L) = \frac{(\bar{n}_d)^n}{(1+\bar{n}_d)^{n+1}}, \tag{S.10}$$

where $\bar{n}_d = \bar{n}\gamma\xi\eta_{\mathrm{pl}}\sin^2\left(\frac{\varphi}{2}\right)$. Additionally, without loss of generality, we describe the initial state ρ before conditional measurements as

$$\rho = \sum_{n=0}^{\infty}\mathrm{p}_{\mathrm{pl}}(n)\sum_{k,l=0}^{n}A_k^n(\xi)A_l^n(\xi)|n-k\rangle\langle n-l|\otimes|k\rangle\langle l|, \tag{S.11}$$

which describes the two-mode state after the reduced plasmonic tritter transformation. We note that this reduced plasmonic tritter transformation is similar to the beam splitter transformation, therefore $A_k^n(\xi) = \sqrt{\binom{n}{k}\xi^{n-k}(1-\xi)^k}$. Finally, the positive-operator-valued measure (POVM) of a realistic photon-counting device with quantum efficiency η is given by [4]:

$$\Pi_L(\eta) = \sum_{m=L}^{\infty}B_{m,L}(\eta)|m\rangle\langle m|, \tag{S.12}$$

in which $B_{m,L}(\eta) = \binom{m}{L}\eta^L(1-\eta)^{m-L}$. Combining the above equations, we have

$$\rho_e(L) = \frac{1}{\mathrm{p}_d(L)}\sum_{m=L}^{\infty}\sum_{n=0}^{\infty}B_{m,L}\left(\eta_{\mathrm{pl}}\right)\mathrm{p}_{\mathrm{pl}}(m+n)\left[A_m^{m+n}(\xi)\right]^2|n\rangle\langle n|. \tag{S.13}$$

Then we can calculate the conditional mean occupation number using Eq. (S.13),

$$\bar{n}_e = \bar{n}\gamma\xi\eta_{\rm ph}\cos^2\left(\frac{\varphi}{2}\right)\left(\frac{L+1}{1+\bar{n}\gamma(1-\xi)\eta_{\rm pl}\cos^2\frac{\varphi}{2}}\right). \tag{S.14}$$

Similarly, one can calculate the standard deviation of the number of detection events of mode $\hat{e}$, when conditioned on the detection of L plasmons,

$$\Delta n_e = \frac{\bar{n}_e}{\sqrt{\frac{(1+L)\bar{n}\gamma\eta_{\rm ph}\xi\cos^2\frac{\varphi}{2}}{1+\bar{n}\gamma(\xi\eta_{\rm ph}+(1-\xi)\eta_{\rm pl})\cos^2\frac{\varphi}{2}}}}. \tag{S.15}$$

Finally, the L-plasmon subtracted signal-to-noise ratio (SNR) is given by

$$\mathrm{SNR} = \frac{\bar{n}_e}{\Delta n_e} = \sqrt{\frac{(1+L)\bar{n}\gamma\eta_{\rm ph}\xi\cos^2\frac{\varphi}{2}}{1+\bar{n}\gamma(\xi\eta_{\rm ph}+(1-\xi)\eta_{\rm pl})\cos^2\frac{\varphi}{2}}}. \tag{S.16}$$

[1] U. Felix-Rendon, P. Berini, and I. D. Leon, Ultrasensitive nanoplasmonic biosensor based on interferometric excitation of multipolar plasmonic modes, Opt. Express **29**, 17365 (2021).

[2] S. M. H. Rafsanjani, M. Mirhosseini, O. S. M. na Loaiza, B. T. Gard, R. Birrittella, B. E. Koltenbah, C. G. Parazzoli, B. A. Capron, C. C. Gerry, J. P. Dowling, and R. W. Boyd, Quantum-enhanced interferometry with weak thermal light, Optica **4**, 487 (2017).

[3] A. Safari, R. Fickler, E. Giese, O. S. Magaña Loaiza, R. W. Boyd, and I. De Leon, Measurement of the photon-plasmon coupling phase shift, Phys. Rev. Lett. **122**, 133601 (2019).

[4] A. Allevi, A. Andreoni, M. Bondani, M. G. Genoni, and S. Olivares, Reliable source of conditional states from single-mode pulsed thermal fields by multiple-photon subtraction, Phys. Rev. A **82**, 013816 (2010).
